# Are palm reversals the pronoun reversals of sign language? Evidence from a fingerspelling task

**DOI:** 10.3389/fpsyg.2022.953019

**Published:** 2022-10-14

**Authors:** Aaron Shield, Megan Igel, Richard P. Meier

**Affiliations:** ^1^Department of Speech Pathology and Audiology, Miami University, Oxford, OH, United States; ^2^Department of Linguistics, University of Texas at Austin, Austin, TX, United States

**Keywords:** autism spectrum disorder, ASL, pronouns, modality, fingerspelling

## Abstract

Acquisition of pronominal forms by children with autism spectrum disorder (ASD) continues to garner significant attention due to the unusual ways that such children produce and comprehend them. In particular, pronoun reversal errors (e.g., using the 2nd-person pronoun “you” to refer to oneself) have been noted in the speech of children with ASD since the very first report of the disorder. In more recent years, investigations of the signing of deaf children with ASD have documented a different phenomenon: palm orientation reversals, such that signs typically produced with an outward-facing palm are produced with the palm towards the signer, or vice versa. At the same time, true pronoun reversals have yet to be documented in the signing of deaf children on the autism spectrum. These two curious facts have led us to ask if there is evidence that palm orientation reversals in signed languages and pronoun reversals in spoken languages could be surface manifestations of the same underlying differences present in ASD. In this paper we seek to establish whether there is evidence for such an analogy, by comparing the ages at which the two phenomena appear in both typically-developing (TD) children and those with ASD, the frequency and consistency with which they appear, and their relationships with other linguistic and cognitive skills. Data are presented from a fingerspelling task given to a sample of 17 native-signing children with ASD and 24 native-signing TD children. We conclude that there are provocative parallels between pronoun reversals in spoken languages and palm reversals in signed languages, though more research is needed to definitively answer these questions.

## Introduction

Over the past decade we have pursued a new line of research investigating the linguistic development of children with autism spectrum disorder (ASD) exposed to a signed language from birth by their Deaf parents; these children are native signers. This work is of theoretical interest because such studies show how children with ASD acquire language in a modality other than speech. As such, they have the potential to shed new light on how language acquisition occurs in ASD, how acquisition is related to and dependent on particular social skills, and how language modality affects acquisition. So far, these studies have documented several phenomena that mirror the development of speaking children with ASD, such as pronoun avoidance (Jordan, 1989; Lee et al., 1994; Shield et al., 2015), difficulties with theory of mind (Shield et al., 2016), articulation challenges (Bhat et al., 2016; Shield et al., 2017), and atypical perception and production of facial expressions (Denmark et al., 2014, 2019). At the same time, one well-documented feature of the spoken language of children with ASD – pronoun reversals (Kanner, 1943; Naigles et al., 2016) – has yet to be clearly documented in signing children with ASD, despite attempts to elicit sign language pronouns (Shield et al., 2015). Complementing this striking absence is the documentation of a different kind of reversal – the reversal of the orientation of the palm in the signing of such children (Shield and Meier, 2012; Shield et al., 2020). Such reversals have also been documented in the imitation of gestures by hearing children with ASD (Ohta, 1987), and have been interpreted as being due to differences in imitation style (Shield and Meier, 2018), difficulties with “self-other mapping” (that is, the ability to faithfully reproduce the body movements of others, Rogers and Pennington, 1991), or with breakdowns in intersubjective identification (Hobson and Hobson, 2007).

Pronoun reversals in speech and palm orientation reversals in sign share a fundamental quality: they both reflect the wholesale or gestalt reproduction of a linguistic form produced by a speaker/signer, as it is perceived by the interlocutor. In the case of pronoun reversals, children typically produce the second-person pronoun (e.g., “you” in English) in reference to self, using the pronoun that others use to refer to the child. In the case of palm orientation reversals, children reproduce signs as they appear from their perspective, rather than reversing what they see in order to faithfully produce the sign. Faithful reproduction of a sign requires that the child produce that sign as it would be produced from the signer’s perspective, not their own. It is important to note that we would not predict signed pronoun reversals to result from such a gestalt imitation style: if the child were to produce the ASL pronoun you as it appears from their own perspective, they would (paradoxically) produce an indexical point towards their own body, with or without contact with the torso, which would approximate the appropriate ASL pronoun me.

Despite the fact that these reversals occur in one linguistic domain in speech (deictic pronouns) and in another domain in sign (articulation of the sign itself), it appears possible that both phenomena could be grounded in the same underlying difference – a tendency to reproduce linguistic forms in a gestalt fashion without undergoing a shift – from *you* to *I* for spoken pronouns and from the addressee’s (i.e., the child’s) perspective on palm orientation to the signer’s perspective on palm orientation.

Given the conspicuous lack, thus far, of documented pronoun reversals in the signing of children with ASD, paired with the clear documentation of palm orientation reversals in the same children, in this paper we ask if there is any evidence that palm orientation reversals in sign could be analogous to pronoun reversals in speech. By analogous, we mean that they have the same underlying causes, despite having different surface forms.

What kind of evidence might be sufficient to prove or disprove such a hypothesis? One way to establish such a connection would be to show that pronoun reversals in speech and palm orientation reversals in sign occur:

(a) at similar chronological ages (for both typical and atypical children);
(b) at similar frequencies and with similar (in)consistency within the population of children with ASD;
(c) in children with similar linguistic and/or cognitive profiles, and/or
(d) in individual children with ASD who are bimodal bilinguals in a signed language and in a spoken language.

If we were to find similarities such as the above, then we might be able to start to build an argument that these could be analogous phenomena – in other words, that the acquisition of language by children with ASD is marked by a similar approach by the learner which, however, results in different surface forms in signed and spoken languages.

In the section that follows, we will briefly lay out what is known about pronoun reversals in speaking children, in order to establish a baseline against which to compare the production of palm orientation reversals by signing children as reported in prior studies and in the current study. We will focus primarily on points (b) and (c) above, with an admittedly incomplete picture regarding point (a), especially with regard to typical children. We do not have data that would address point (d), but suggest that this is a promising avenue for future research.

### Pronoun reversals in speaking children

#### When do pronoun reversals occur?

##### Typical development

First-and second-person forms (*I, me, you*) typically emerge around the age of 1;6 or when children’s MLU reaches 2.5 (Bloom et al., 1975). A number of studies have documented that typically-developing (TD) hearing children sometimes reverse first-and second-person pronouns early on in development, generally before the age of 2;6. Dale and Crain-Thoreson (1993) found that 17 of 30 precocious speakers reversed pronouns at age 1;8. Several case studies have also documented pronoun reversals in very young TD children, especially early talkers. Evans and Demuth (2012) found that one TD child reversed pronouns from age 1;7 to 2;4; Chiat (1982) reported a TD child who reversed both first-and second-person pronouns between ages 2;4–2;5; and Oshima-Takane (1992) discussed another TD child who produced reversed pronouns between 1;11 and 2;4.

In the acquisition of signed languages, there are indications that some very young TD signing children also go through a phase of pronoun reversal between the ages of 1;3–2;0 (Petitto, 1987; Jackson, 1989; Pizzuto, 1990), despite the phonological similarity of sign-language pronouns to gestural points, which typically emerge by 8–10 months (Bates and Dick, 2002). Such errors have been interpreted as the child’s treatment of the indexical point as a frozen lexical form, such that a point away from the child’s body toward an interlocutor (thereby producing a sign that looks like the sign you) is meant to refer to the child (“me”). Thus, in both hearing and deaf TD children, pronoun reversals most often occur before age 2;6, and appear to be the result of linguistic development that has outpaced the social or pragmatic abilities necessary to use such forms in an adult way.

##### Atypical development

For children with ASD, reversals have also been found to start early, but often persist well past the age of 2;6. Evans and Demuth (2012) found that reversals started at 1;5 and continued through the end of data collection at age 2;11 in their single subject with ASD. Naigles et al. (2016) studied 15 children with ASD between the ages of 2;6 and 4;6, finding reversed pronouns in older children as well as younger children.

Several studies attest to continued pronoun reversal by children with ASD well into the school-age years and even adolescence. In the first modern report of ASD, Kanner (1943) observed that 7 of 11 children ranging in age from 3;6 to 6 years reversed or confused pronouns.^1^ Tager-Flusberg (1994) found that six children with ASD between the ages of 3;4 and 9;9 produced 220 reversed pronouns (13.2% of the total pronouns in the corpus). Jordan (1989) found that three children with ASD reversed pronouns in a sample of 11 children and adolescents with ASD between the ages of 6;8 and 16;5, and an MLU between 1.1–4.8 (*M* = 2.4). Finally, Lee et al. (1994) found that three of 25 adolescents with ASD ages 14–17 made pronoun reversal errors, producing “I” instead of “you.”

Thus, pronoun reversals are attested in children with ASD beyond the age at which they typically disappear (~2;6), into later childhood and even into adolescence.

#### How frequently or consistently do pronoun reversals occur?

##### Typical development

Most studies of pronoun development in TD children report infrequent pronoun reversals (Loveland, 1984; Dale and Crain-Thoreson, 1993; Evans and Demuth, 2012; Naigles et al., 2016), both with respect to the percentage of children who produce the reversals, and the percentage of pronouns that are reversed by such children. For example, Loveland (1984, p. 548) reported pronoun reversals in a group of 11 TD children aged 2;0–2;3, but “no children in this study were observed to make frequent or consistent pronoun-production errors of the reversal type.” More recently, Naigles et al. (2016) found that 1.67% of pronouns produced by TD children were reversed between 1;9–2;3, which decreased to under 1% of pronouns between 2;9–3;3. Evans and Demuth (2012) reported that their TD participant reversed 3% of 1st-person pronouns, but 79% of 2nd-person pronouns between 0;11 and 2;6. Rarely, some children consistently reverse pronouns (e.g., Oshima-Takane, 1992) for a period of time before they learn the correct use of the pronominal system. Dale and Crain-Thoreson (1993, p. 576) observed, “cases where children consistently reverse pronouns (such as Oshima-Takane’s subject) seem relatively rare. More typical is an intermittent, low frequency pattern of errors.” Thus, when pronoun reversals occur in typical development, they are usually inconsistent and occur at a low frequency.

##### Atypical development

Most studies have found that speaking children with ASD reverse pronouns at a higher frequency than TD children, though the specific frequencies found by individual scholars have varied. With respect to the percentage of hearing children with ASD who produce pronoun reversals, studies have ranged on the low end from just one of 38 children (2.6%) with ASD at age 4 (Barokova and Tager-Flusberg, 2020), to 7 of 11 such children (63.6%; Kanner, 1943) on the high end, with other reports falling somewhere in the middle: Lee et al. (1994) reported reversals in 3 of 25 adolescents (12%) with ASD ages 14–17, while Jordan (1989) reported reversals in 3 of 11 children (27.3%) with ASD between the ages of 6;8–16;5.

Shield et al.’s (2015) study is the only study to-date on signed pronouns produced by signing children with ASD. This study had both a naturalistic and an elicited (experimental) component. ASL pronouns produced during naturalistic observation were analyzed in their discourse contexts by independent raters in order to identify potential pronoun reversals. Two possible examples were identified, both in echolalic contexts. In neither case was it clear that the child had intended to refer to either himself or the investigator, as he tended to echo most utterances and had very low receptive language skills overall. In the elicited pronoun task of the same study, none of the 15 native-signing children with ASD from whom ASL pronouns were elicited produced any reversed forms, suggesting that pronoun reversals in ASL may not occur as frequently as they do in spoken languages.

In studies that were either case studies or reported total frequency of pronoun reversal across the samples, we also find a range of frequencies. Naigles et al. (2016) reported that the 15 children with ASD in their sample reversed 6.4% of pronouns between 2;6–3;6, which decreased to 4.15% of pronouns between 3;9–4;6. Several other studies have found higher frequencies of pronoun reversals: Evans and Demuth (2012) reported that their case-subject with ASD reversed 13% of 1st-person pronouns and 79% of 2nd-person pronouns between ages 0;11 and 2;11, while Tager-Flusberg (1994) found that 13.2% of all personal pronouns were reversed by six children with ASD ages 3–10 years.

Thus, most studies have found that, when children with ASD produce pronoun reversals, they do so at relatively low frequencies, and are rarely consistent in producing reversals. In comparison with TD children, children with ASD appear to produce a higher rate of pronoun reversals (e.g., 6.4% of total pronouns at ages 2;6–3;6 for children with ASD compared to <1% of total pronouns between 2;9–3;3 for TD children; Naigles et al., 2016).

#### Which cognitive skills are implicated in the production of pronoun reversals?

##### Typical development

Several studies have found that pronoun reversals are produced by TD children when their language development has outpaced their social, cognitive, or pragmatic development. Evans and Demuth (2012) attributed pronoun reversals to precocious talkers who had not yet mastered the deictic (perspective-taking) nature of the pronominal system. Petitto (1987) had a similar interpretation of the two signing children she observed, who seemingly treated indexical points as frozen lexical signs, echoing Clark’s (1978) hypothesis that very young children may assume that pronouns function like names with fixed referents. Similarly, Dale and Crain-Thoreson (1993, p. 581) observed that their pronoun “reversers appear to be somewhat more advanced grammatically [than non-reversers]: their grammatical morpheme index is significantly higher, and their MLU is higher, though non-significantly, than those of the non-reversers.” The development of social-cognitive skills such as perspective-taking and theory of mind (ToM) have been shown to support the proper use of pronouns: for example, Loveland (1984) found that children who showed evidence of perspective-taking ability did not reverse pronouns. In line with these studies, Overweg et al. (2018, p. 228) concluded that ToM understanding “was associated with correct pronoun interpretation in older TD children relative to younger TD children, … indicat[ing] that pronoun reversals most likely result from perspective-shifting difficulties.” Finally, some have theorized that pronoun reversals could result from heavy cognitive load in complex situations, even when children understand perspective-taking (Dale and Crain-Thoreson, 1993). Thus, when pronoun reversals occur in typical development, they appear to result from a mismatch between the rate of development of language and the social or cognitive skills that are needed to understand and produce deictic forms.

##### Atypical development

Pronoun reversals in children with ASD have been attributed to various causes, including echolalia, delayed language development, intellectual and cognitive deficits, and pragmatic difficulties. Kanner (1943) believed that pronoun reversals were the result of echolalia, and others have made similar claims, such as that reversed pronouns are produced because children with ASD repeat rote phrases they have heard from others (Ricks and Wing, 1975). Unlike precocious TD children, delayed language development has been implicated in the production of pronoun reversals by children with ASD (Tek et al., 2014), specifically low MLU (Chiat, 1982; Loveland and Landry, 1986; Dale and Crain-Thoreson, 1993) or syntactic difficulties (Tager-Flusberg, 2006; Eigsti et al., 2007). Other reports find a connection with intellectual disability (Kanner, 1943; Tager-Flusberg, 1994), perspective-taking skills involving theory of mind (Meir and Novogrodsky, 2019), or difficulties with pragmatics, specifically understanding how pronominal forms shift reference between speakers in discourse (e.g., Charney, 1980; Hobson, 1990; Tager-Flusberg, 1996; Hobson et al., 2010; Mazzaggio and Shield, 2020). Pronoun reversals may also arise through the interaction of multiple factors in development, specifically when language outpaces social development (Evans and Demuth, 2012). For example, Naigles et al. (2016) found that children with ASD who produced more pronoun reversals than TD children also had lower joint attention scores, whereas children with ASD who had higher vocabulary and joint-attention scores produced fewer pronoun reversals in imitative contexts, thus implicating both language and social abilities in producing pronoun reversals.

In sum: pronoun reversals are produced by very young TD children (usually before 2;6) and older children with ASD into adolescence; they are produced relatively infrequently, accounting for under 10% of pronouns produced by children with ASD, and they are produced by children whose social cognition lags behind their language development, or by children with echolalia or language impairment.

In the next section we will review what is currently known about the occurrence of palm orientation reversals in signing children.

### Palm orientation reversals in signing children

To date, there are two reports of palm orientation reversals produced by signing children with ASD: Shield and Meier (2012) studied five native-signing children with ASD (four deaf children and one hearing child of Deaf adults) ranging in age from 4;6 to 7;5 and Shield et al. (2020) published a longitudinal case study of a single native signer with ASD over the span of 10 years, from age 4;11 to 14;11.

Shield and Meier (2012) described two studies: naturalistic observation and elicited fingerspelling. During observation of spontaneous interactions between three children with ASD and their Deaf parents, Child 1 (age 7;5) produced 50 fingerspelled letters with the palm orientation facing inward rather than outward. Child 2 (age 4;6) produced three lexical signs (the number signs six, seven, and eight) with inward palm orientation rather than outward, and Child 3 (a hearing children of deaf adults aged 6;6) produced the handwave gesture and the lexical sign flashing-light with an inward rather than outward palm orientation. The fingerspelling task looked at four native-signing children with ASD; three of these children (ages 5;8, 6;6, and 7;5) reversed the palm orientation of 72 of 179 (40.2%) fingerspelled letters such that the children’s palm faced toward their own body rather than outward. None of the control group of 12 typical deaf children (ages 3;7–6;9) produced any such palm orientation reversals. The three children with ASD who made such errors had lower parent-reported language scores on the Language Proficiency Profile-2 (LPP-2; Bebko et al., 2003) than those children who did not make such errors, including the 12 typical deaf children and the child with ASD who did not make any palm reversals. This significant difference suggests that children with lower receptive and expressive language skills may be more prone to making such errors.

In the later case study, Shield et al. (2020) described the signing of a single native-signing child with ASD, a left-handed hearing male who is the child of two Deaf parents. They analyzed his signing at ages 4;11, 6;6, 10;2, and 14;11, reporting that while his signing improved consistently in terms of handshape, location, and movement, the error rate in palm orientation remained high, reaching over 50% of all signs produced at age 14;11. They distinguished between midline errors (i.e., palm orientation errors in which the palm is oriented toward the midline rather than facing inwards or outwards), which could be attributed to motor challenges (since the palms face the midline in the resting position of the arms), and 180-degree reversal errors, which are unlikely to be produced due to motor issues and are more likely due to differences in imitation. The child produced a total of 82,180-degree reversal errors over the four data collection sessions (one at age 4;1, 15 at age 6;6, 8 at age 10;2, and 58 at age 14;11); all but five of these reversals were produced on fingerspelled letters, with the remainder being produced on lexical signs. For this child as well as the children described in Shield and Meier (2012), the palm reversals on lexical signs cannot be attributed to coarticulation effects because the signs were produced in isolation as single signs. Even at age 14;11, the participant produced 180-degree reversal errors on 58 of 112 total palm orientation errors (51.8%), providing the first indication that palm orientation errors can persist into adolescence for some signers with ASD.

Thus, there is evidence that some children with ASD produce palm orientation reversals, while TD signing children do not appear to do so, at least not at the ages studied. Furthermore we have preliminary indication that such reversals can persist into adolescence. However, what is currently unknown is how frequently such reversals tend to occur, at what ages, whether or not they occur in typical development, and if signers who produce such reversals share a particular linguistic or cognitive profile. Such information would be useful in order to establish a comparison between palm reversals and pronoun reversals. However, we should caution from the outset that, given the wide age ranges and relative infrequency of both phenomena, our conclusions must be considered preliminary. Still, a clearer characterization of the palm reversal phenomenon in particular would help bring potential comparisons into focus.

In order to better understand the occurrence of palm orientation reversals in child development, the study that follows probes the frequency with which palm orientation reversals are produced by signing children with and without ASD. The study will help us to understand the cognitive and linguistic profiles of children who produce such reversals, and whether or not palm reversals are appropriately considered a sign-language analog to pronoun reversals in speech.

## Materials and methods

### Participants

The participants in this study have been described in several prior publications (Shield et al., 2015, 2016, 2017; Bhat et al., 2016); however, the tasks described in this paper have not previously been analyzed for palm orientation. For the current study, we included two groups of participants: (1) native-signing children with ASD (*N* = 17; four females; age range 5;0–14;4; mean age 9;10) and (2) a control group of native-signing Deaf children who are typically-developing (*N* = 24; 14 females; age range 6;1–12;9; mean age 8;10). All of the children were themselves deaf except for two hearing children of Deaf adults in the ASD group, participants M7 and M17.

Three of the children who participated in Shield and Meier’s (2012) preliminary fingerspelling study reported on above also participated in this study (approximately 5 years later). Child 1, aged 7;5 in the earlier study, is referred to here as M8, and was tested at age 12;7; Child 3, aged 6;6 in the earlier study, is referred to here as M7, and was tested at age 10;2; and Child 4, aged 5;8 in the earlier study, is referred to here as M4, and was tested at age 9;8.

### Assessments

All participants were administered a battery of tests in order to gather information regarding their nonverbal intelligence, linguistic abilities, and social skills. In order to assess nonverbal intelligence, the Test of Nonverbal Intelligence, Fourth Edition was administered (TONI-4; Brown et al., 2010). To assess receptive competence in ASL, the American Sign Language Receptive Skills Test (ASL RST; Enns et al., 2013) was administered. The TONI-4 and ASL RST use standard scores (SS), which have a mean score of 100 and a standard deviation of 15. Scores between 85 and 115 are considered to lie within normal limits.

ASD diagnosis was confirmed *via* the Autism Diagnostic Observation Schedule, Second Edition (ADOS-2; Lord et al., 2012). Only the participants with ASD were administered the ADOS-2. The Social Communication Questionnaire (SCQ; Rutter et al., 2003) was completed by the parents of all participants in order to ensure that participants in the control group were not above threshold for ASD risk.

Finally, two experimental tests were administered in order to assess social competence. A minimally-verbal test of theory of mind (ToM), specifically false-belief, involved the participants being given picture cards sequenced to tell a story based on Wimmer and Perner's (1983) unseen-displacement task. Participants were tasked with identifying the appropriate ending from a choice of two picture cards (as described in Shield et al., 2016). A minimally-verbal test of visual perspective-taking (VPT) tasked participants with matching their own perspective or the perspective of the experimenter, who was seated across the table, to a three-dimensional toy on a turntable between them (as described in Shield et al., 2016). ToM and VPT were measured in four trials each and reported as overall accuracy proportions out of four, with overall scores ranging from zero to one. The scores on each of these assessments for all participants are reported in Table 1 below.

**Table 1:** Group mean scores and standard deviations on assessments.

Group	Age (SD)	TONI SS (SD)	ASL RST SS (SD)	SCQ (SD)	ToM (SD)	VPT (SD)
ASD (N = 17)	9.82 (2.76)	96.94 (12.18)	90.71 (12.36)	14.29 (6.80)	0.58 (0.37)	0.30 (0.44)
TD (N = 24)	8.86 (1.83)	103.92 (12.09)	109.29 (6.73)	2.67 (2.73)	0.82 (0.24)	0.64 (0.43)
*p*-value	0.60	0.08	** < 0.001	** < 0.001	*0.03	*0.02

*p<0.05 and **p<0.001.

Although the children in the two groups did not differ statistically in chronological age or nonverbal intelligence, the groups differed significantly in receptive language abilities, ToM, and VPT. Specifically, the TD group had significantly higher receptive language, ToM, and VPT scores. The ASD group had significantly higher SCQ scores than the control group, and all of the TD participants scored under the threshold score for ASD risk on the SCQ (=11).

### Procedure

**Figure 1:**
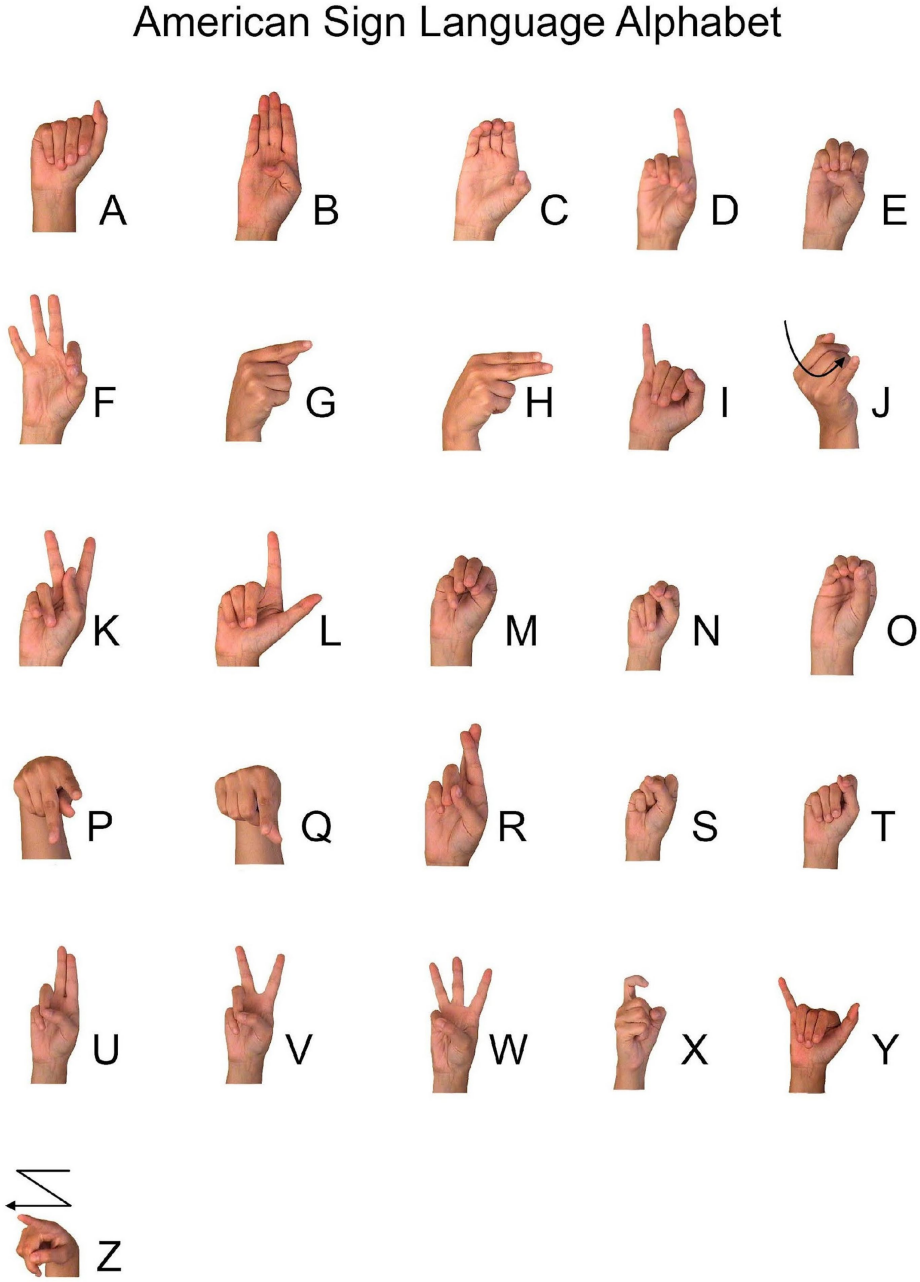
Handshapes of the ASL Fingerspelling Alphabet. ASL alphabet image freely available from StartASL.com, copyright © 2008 StartASL.com.

We used a fingerspelling task to elicit signs because palm orientation errors have surfaced most often in fingerspelling although such errors have also been documented in lexical signs (Shield and Meier, 2012). For example, fingerspelling accounted for 110 of the 112 (98.2%) palm orientation errors produced by the child described by Shield et al. (2020) at age 14;11. Fingerspelled letters are produced in neutral space in front of the signer’s body and, with the exception of the letters g, h, p, and q, are typically produced with the palm of the signer facing outward towards an interlocutor; see Figure 1. Thus, fingerspelled letters provide many opportunities for reversal, having a specified palm orientation (outward for all letters except g and h, which face inward, and p and q, which face downward) and lacking an anchor to the signer’s body, which could attenuate reversal.

Deaf parents sometimes include fingerspelled words in their signing to their very young deaf children (e.g., as early as 2 months old; Kelly, 1995), and sign-exposed children learn to fingerspell very early on, with some children producing fingerspelled words as early as age two (Kelly, 1995; Erting et al., 2000). Padden (1991) has explained that deaf children “learn to fingerspell twice”: that is, they first learn to produce fingerspelled words as if they were lexical signs, and later they learn to connect these signs to written English words. Thus, the ability to fingerspell emerges naturally as children acquire ASL, but only later is fingerspelling explicitly linked to written representations. As all of the children in our study were school-age, we determined that presenting written English words as stimuli for fingerspelling would be an appropriate format.

The following lowercase written words were presented by the lead author, a hearing late learner of ASL, to each of the participants on a tablet: *ball, paper, girl, school, bird, teach, phone, desk, chair, table, doll, father, mother, van,* and *bug*. Thus, participants had the opportunity to produce 69 individual fingerspelled letters in these English target words. The participants were presented with each of the stimulus words one at a time and were instructed to fingerspell each word that appeared on the screen. The participants were able to view the written English words on the tablet screen while fingerspelling, thus eliminating any demands on working memory. Once the word was fingerspelled, the investigator presented the next word. Participants completed this task independently without feedback; any deviations from accurate spelling or correct handshape production were not corrected by the investigator.

### Coding

Using ELAN (EUDICO Linguistic Annotator; Tacchetti, 2017) multimodal coding software, each handshape produced was coded for its alphabetic label (a, b, c, etc.) and the palm orientation of each signed letter was coded as *inward* (facing the signer’s body), *outward* (facing away from the signer’s body), *upward* (facing the ceiling), *downward* (facing the floor), or *midline-facing* (facing toward the signer’s midline; i.e. towards the left for a right-handed signer or towards the right for a left-handed signer). Each palm orientation value was scored as being produced *correctly* or as an *error* based on standard citation forms. Errors were classified as *reversal errors* (substitutions of inward orientation for outward and vice versa), *midline errors* (for midline-facing orientations), or *other errors* (upward or downward orientations, except for p and q, which have downward-facing orientations in their citation forms).

While producing the fingerspelled letters c and o with a midline-facing orientation is widely accepted within signing communities, for the sake of consistency, these letters were coded as *midline* errors if produced midline-facing. Similarly, the production of P with a midline-facing or even a slightly inward-facing palm orientation reflects variation seen among native signers (Geer, 2016). For the purposes of our analyses, these errors were coded as *midline* errors for midline-facing productions, or *other* errors for inward-facing productions, but were not coded as palm reversal errors, as these variants are used among native signers (Geer, 2016).

In addition to palm orientation errors, we also coded how accurately the participants were able to spell the written word (i.e., spelling errors). Fingerspelled letters were coded as spelling errors if the handshape produced represented a letter that does not appear in the target English word or if it was produced in a different order from the target English word. False starts (e.g., c-h-c-h-a-i-r for “chair”) were not coded as errors if the word was ultimately spelled correctly; neither were double/single letters (e.g., d-o-l for “doll”) coded as errors since it is acceptable in ASL fingerspelling to produce a double letter just once, with a slight hold.

### Reliability

To ensure the reliability of the coding system, each video was coded by a second and third trained coder experienced in the coding of ASL. Differences in coding were discussed by the coders and disagreements were resolved through consensus. The main coder then adjusted the rest of the coding to reflect the decisions made through consensus discussion with the additional coders.

## Results

We examined all of the fingerspelled letters produced by both groups and calculated the number of letters that were produced with the three kinds of palm orientation errors. The total number of fingerspelled letters produced by the two groups differed because there were different numbers of children in each group and because individual children produced different numbers of fingerspelled letters, usually due to spelling errors or repeated fingerspelling attempts. All fingerspelled letters were coded, regardless of the number of times the child attempted to spell the target word.

**Figure 2:**
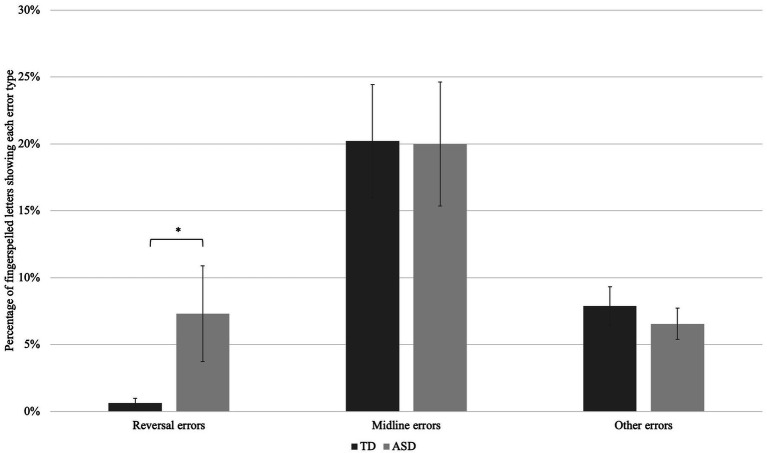
Mean error rates, by group, for three types of palm orientation error. Groups did not differ in their rate of production of midline or other errors, but the ASD group made significantly more reversal errors than the TD group, *p* = 0.05.

The TD group produced a total of 1742 fingerspelled letters, whereas the ASD group produced 1,191. TD children produced an average of 72.6 (*SD* = 7.46) letters whereas the children with ASD produced an average of 70.1 letters (*SD* = 15.8); this difference was not significant; *t* (39) = 0.69, *ns.* Note that one very young child with ASD (M9, age 5;3) did not complete the task and only produced 12 fingerspelled letters. The ASD group produced more spelling errors (total = 110; *M* = 6.5, *SD =* 5.8) than the TD group (total = 39; M = 1.6, SD = 2.9), *t* (39) = 3.54, *p* = 0.001. The ASD group also produced more palm reversal errors (*M* = 5.12, *SD* = 11.34) than the control group (*M* = 0.46, *SD* = 1.25), *t* (39) = 2.02, *p* = 0.05. TD children produced an average of 14.67 midline errors (*SD* = 15.89) whereas the children with ASD produced an average of 14.0 midline errors (*SD* = 13.7); this difference was not significant; *t* (39) = 0.14, *ns*. TD children produced an average of 5.71 other errors (*SD* = 4.91) whereas the children with ASD produced an average of 4.59 other errors (*SD* = 3.74); this difference was not significant; *t* (39) = 0.65, *ns*. See Figure 2 for a comparison of the error rates for the three error types. Spelling accuracy was weakly related to the production of palm reversal errors; *r* (39) = 0.33, *p* < 0.05.

Since the two groups did not differ in their rate of production of midline or other errors, we next examine the reversal errors produced by children from the two participant groups.

### Typically-developing children

Six of the 24 participants in the control group of TD deaf children produced at least one palm reversal error. Five of these six children produced a single error, while one child (F7) produced six palm reversal errors. The palm reversal rate for each TD participant is shown in Table 2 below.

**Table 2:** Typically-developing children: Palm reversal rates on fingerspelled letters.

Participant ID	Age (years; months)	*N* of palm reversals/Total fingerspelled letters	Reversal rate
F1	8;7	0/66	0.0%
F2	7;7	0/67	0.0%
F3	7;7	0/79	0.0%
F4	10;3	1/53	1.9%
F5	9;7	0/69	0.0%
F6	6;7	0/73	0.0%
F7	6;6	6/74	8.1%
F8	11;2	0/86	0.0%
F9	11;6	0/75	0.0%
F10	7;7	0/68	0.0%
F11	7;7	1/76	1.3%
F12	8;9	0/74	0.0%
F13	8;5	0/77	0.0%
F14	9;3	1/67	1.5%
M1	8;10	0/84	0.0%
M2	7;9	0/73	0.0%
M3	9;11	0/68	0.0%
M4	9;7	1/69	1.4%
M5	8;7	0/77	0.0%
M6	12;2	0/77	0.0%
M7	12;9	0/69	0.0%
M8	9;11	0/65	0.0%
M9	6;1	0/69	0.0%
M10	6;3	1/87	1.1%
		Total = 11/1742	0.6%

Six of the 11 (55%) palm reversal errors made by the control group, all produced by participant F7, were in-to-out reversals, meaning a letter with a citation form palm-in orientation was produced with an inaccurate palm-out orientation; all were instances of the letter h, which the child produced like the letter u every time the target letter h appeared in a word (with outward-facing palm orientation and fingers oriented vertically rather than horizontally; see Figure 1).^2^ The five remaining errors produced by this group were out-to-in errors: two errors on fingerspelled letters directly following the palm-in letter h, one error on the letter e in a word (*phone*) containing the palm-in letter h and two on the letters a and r in the word *paper*. The palm reversal errors produced by this group are found in Table 3 below.

**Table 3:** Typically-developing children: Fingerspelling errors.

Stimulus	Participants					
	F4	F7	F11	F14	M4	M10
ball	B-A-L-L-B-A-L	B-A-L-L	B-A-L-L	B-A-L	B-A-L-L	B-A-C-L-L
paper	–	P-A-P-E-R	P-**A**-P-E	P-A-P-E-R	P-A-P-E-**R**	P-A-P-E-P-A-P-E-R
girl	–	G-I-R-L	G-I-R-L	G-I-R-L	G-I-R-L	G-I-R-L
school	–	S-C-**H**-O-O-L	S-C-H-O-O-L	S-C-H-O-L	S-C-H-O-O-L	S-C-H-O-O-L
bird	–	B-I-R-D	B-I-R-D	B-I-R-D	B-I-R-D	B-I-R-D
teach	T-E-A-C-H	T-E-A-C-**H**	T-E-A-C-H	T-E-A-C-H	T-E-A-C-H	D-L-T-O-E-A-C-H
phone	P-H-O-N-E	P-**H**-O-N-E	P-H-O-N-E	P-H-O-N-E	P-H-O-N-E	P-H-O-N-**E**
desk	D-E-S-K	B-D-E-S-K	D-E-S-K-D-E-S-K-D-E-S-K	D-E-S-K	D-E-S-K	D-E-S-D-K
chair	C-H-**A**-I-R	C-**H** A-L-R	C-H-A-I-R	C-H-A-I-R	C-H-A-I-R	C-H-A-H-A-I-R
table	T-A-B-L-E	T-A-B-L-E	T-A-B-L-E	T-A-B-L-E	T-A-B-L-E	T-A-B-L-E
doll	D-O-L-L	B-O-L-L	D-O-L-L	D-O-L-L	D-O-L-L	B-O-O-L-B-O-O-L
father	F-A-T-H-E-R	R-T-E-F-A-T-**H**-E-R	F-A-T-H-R	F-A-T-H-**E**-R	F-A-T-H-E-R	F-A-T-H-E-R
mother	M-O-T-H-E-R	M-O-T-**H**-E-R	M-O-T-H-E-R	M-O-T-H-E-R	M-O-T-H-E-R	M-O-L-T-H-E-R
van	V-A-N	V-A-N	V-A-N	V-A-N	V-A-N	V-W-V-A-N
bug	B-U-G	B-N-U-G	B-B-U-G	B-U-G	B-U-G	D-U-G
Total fingerspelled letters	53	74	76	67	69	87
Total palm reversal errors	1 (1.9%)	6 (8.1%)	1 (1.3%)	1 (1.5%)	1 (1.4%)	1 (1.1%)

Letters produced with a 180-degree palm reversal error are bolded. Letters whose citation-form palm orientation is palm-in are underlined.

### Participants with ASD

Seven of the 17 participants in the ASD group made at least one palm reversal error, and five of these seven produced two or more palm reversal errors. The palm reversal rate for each participant with ASD is shown in Table 4 below. Three of these participants accounted for the preponderance of the total palm reversals (M7, M8, and M17: 79/87 errors). Five of the 87 palm reversal errors (6%) were in-to-out errors, produced on the two letters whose citation-form palm orientation is palm-in, specifically the letters g (3 tokens) and h (2 tokens). The remaining 82 palm reversal errors (94%) were out-to-in errors. The details of each palm reversal error can be found in Table 5 below.

**Table 4:** Children with ASD: Palm reversal rates on fingerspelled letters.

Participant ID	Age (years; months)	*N* of palm reversals/Total fingerspelled letters	Reversal rate
F1	14;4	0/69	0.0%
F4	13;3	1/68	1.5%
F5	9;6	0/75	0.0%
F6	11;1	0/86	0.0%
M1	8;5	1/80	1.3%
M2	9;5	4/71	5.6%
M3	11;3	0/69	0.0%
M4	9;8	0/69	0.0%
M5	9;6	0/78	0.0%
M6	9;0	0/68	0.0%
M7	10;2	10/75	13.3%
M8	12;7	34/79	43.0%
M9	5;3	0/12	0.0%
M10	11;10	2/70	2.9%
M12	5;1	0/73	0.0%
M17	12;6	35/75	46.7%
M19	5;0	0/74	0.0%
		Total = 87/1191	6.7%

**Table 5:** Children with ASD: Fingerspelling errors.

Stimulus	Participant						
	F4	M1	M2	M7	M8	M10	M17
ball	B-A-L-L	B-A-L-L	B-A-L-L	B-A-L-L	B-A-E-L	B-A-L-L	**B-A-L-L**
paper	P-A-P-E-R	P-A-P-E-R	P-A-P-E-R	P-A-P-E-**R**	P-A-P-E-R	P-A-P-E-R	P-**A**-P-**E**-**R**
girl	G-I-R-L	G-I-R-L	**G**-I-R-I	G-I-R-L	G-I-I-R-L	**G**-I-E-R-L	G-I-R-L
school	S-C-H-O-L	S-C-H-O-O-L	S-C-N-O-O-I	S-H-**C-**H-O-O-L	S-C-U-H-O-O-L	S-C-O-O-L	E-**S**-**C**-H-O-O-**L**
bird	B-I-R-D	B-I-R-D	B-I-R-B	B-I-R-D-L	D-I-R-D	B-I-R-D	**B-I-R-D**
teach	T-E-A-C-H	T-E-A-C-H	N-T-E-A-C-N	T-E-T-E-A-**C**-H	C-T-E-A-C-U-H	T-E-A-C-H	**T-E-A-C**
phone	P-H-O-N-E	P-**H**-O-N-E	P-R-N-O-N-E	P–P-H-**O**-**N**-**E**	Q-H-O-N-A	P-H-O-N-E	G-P-H-O-N-E
desk	D-D-E-S-K	D-E-S-K	B-E-S-K	D-E-S-K	**D**-**E**-**S**-**K**	D-E-S-K	D-**E**-**A**-**S**-**K**
chair	C-H-A-I-R	C-H-A-C-H-A-I-R-C-H-A-I-R	C-N-A-I-R	C-H-**A**-I-R	C-H-**A-R-D-I-R**	C-H-A-I-R	C-H-A-**I-R**
table	T-A-B-L-E	T-A-B-L-E	T-A-B-I-E	T-A-B-L-E	**T-A-D-I-L-E**	T-A-B-L-E	**T-A-B-I-T-A-B-L-E**
doll	D-O-L-L	D-O-L-L	B-O-I-I	D-O-L	**D**-O-**L**	D-O-L-L	D-O-L-L
father	F-A-T-H-E-R	F-A-T-H-E-R	F-A-T-N-E-R	D-F-A-T-H-E–**E**-R	**R-F-A-T**-H-**E-R-R**	F-A-T-**H**-E-R	F-**A**-**T**-H-E-R
mother	M-O-T-E-**R**	M-O-T-H-E-R	M-O-T-N-E-R	M-O-**T**-H-**E**-R	**A**-O-**T**-H-**E**-**R**	M-O-T-H-E-R	M-O-T-H-E-R
van	V-A-N	V-A-N	**V**-A-N	W-V-A	**V-A-N**	V-A-N	V-A-N
bug	B-U-G	B-G-U-B-U-G	B-**U**-**G**	B-U-G	**B-U-A**-G-H	B-U-S-G	B-U-G
Total fingerspelled letters	68	80	71	75	79	70	75
Total palm reversal errors	1 (1%)	1 (1%)	4 (6%)	10 (13%)	34 (43%)	2 (3%)	35 (47%)

Letters produced with a 180-degree palm reversal error are bolded. Letters whose citation-form palm orientation is palm-in are underlined.

### Cognitive and linguistic profile of children who reverse

Six of the TD children produced one or more palm reversals, with five producing just a single fingerspelled letter with reversed palm orientation. The five TD children who produced a single palm reversal did not differ from the 18 TD children who produced no palm orientation reversals in chronological age, non-verbal intelligence, ASL receptive language skills, or SCQ scores. However, the one TD child who produced 6 palm orientation reversals (F7) had an SCQ score of 10, just under the threshold score for ASD risk of 11. All other TD children had scores of 7 or under, indicating low risk of ASD.

**Figure 3:**
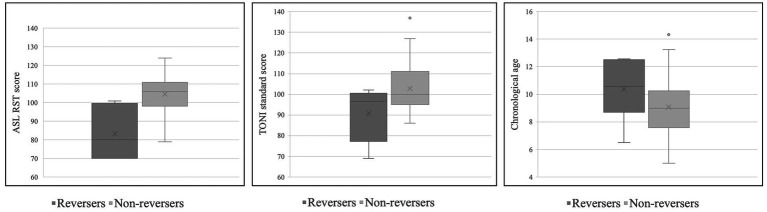
Box-and-whisker plots for reversers and non-reversers in terms of ASL RST scores (left), TONI standard scores (center), and chronological age (right).

Given that in the TD group there were five TD children who produced just one palm orientation reversal, we classified the participants with ASD who produced two or more palm reversal errors as “reversers,” in contrast with those 12 participants with ASD who produced zero or one palm reversal errors (“non-reversers”). Reversers had lower overall receptive language abilities (as measured by the ASL RST) than non-reversers, *t* (15) = −2.81, *p* < 0.05. The reversers and non-reversers did not differ significantly in age, nonverbal intelligence, ASD severity (as indicated by ADOS-2 or SCQ scores), theory of mind, or visual perspective-taking, though note that the TONI (non-verbal IQ) scores of the reversers were nominally lower than the non-reversers, and the reversers were nominally older than the non-reversers. Group means are reported in Table 6 below. We also include box-and-whisker plots of the reversers and non-reversers in terms of ASL RST scores, TONI standard scores, and chronological age in order to better visualize the distribution of data for the two groups (Figure 3).

**Table 6:** Children with ASD: Characteristics of reversers versus non-reversers.

Group	Age	TONI	ASL RST	ADOS severity	SCQ	ToM	VPT
Reversers (*N* = 5)	11.13 (1.40)	89.40 (14.31)	79.80 (11.86)	6.60 (1.95)	15.40 (10.36)	0.40 (0.45)	0.40 (0.55)
Non-Reversers (*N* = 12)	9.28 (3.04)	100.08 (10.23)	95.25 (9.72)	5.18 (2.60)	13.83 (5.24)	0.66 (0.32)	0.25 (0.40)
*p*-value	0.11	0.18	*0.04	0.25	0.76	0.29	0.60

*p<0.05.

### Phonetic context of reversals

Shield et al. (2020) posited that palm orientation errors could be rooted in motoric factors or in differences in imitation strategy. They established that fingerspelled letters oriented towards the midline (rather than clearly outward or inward) could be the result of underarticulation, and thus motoric in origin. Both TD children and children with ASD in this study produced midline palm orientation errors, as shown in Figure 2.

Shield et al. further hypothesized that palm orientation reversals produced during spontaneous signing could reflect the effects of a “visual matching” imitation strategy employed during learning in which the child produces signs as they appear from the child’s perspective. However, it is also possible that some palm reversals could be due to coarticulation; that is, due to adjacency to another fingerspelled letter with the opposite palm orientation. As laid out above, there are four fingerspelled letters in the ASL alphabet that differ in palm orientation from all of the others: g and h (which have an inward-facing palm orientation) and p and q (which face downward). All other fingerspelled letters face outward from the signer. We thus examined the phonetic context in which reversal errors were produced in order to determine if coarticulation could be responsible for the reversals.

For the TD participants, we exclude subject F7’s six productions of h as u, inasmuch as she produced this form in every word that included the letter “h.” Of the remaining five reversals, three occurred immediately after the letters p or h: subject F11 reversed the letter a in “paper”; subject F4 reversed the letter a in “chair,” and subject F14 reversed the letter e in “father.” It is plausible that each of these reversals occurred due to assimilation to the palm orientation value of the previous handshape. The other two reversals produced by TD children occurred word-finally: the letter r in “paper” by subject M4 and the letter e in “phone” by subject M10. Although the motivation for these reversals is less clear, each of the reversals occurred in words in which the letters p or h also appeared, raising a question of whether the palm orientation specification could spread across non-adjacent segments.

For the participants with ASD, 12 of the 87 palm reversals can be explained by adjacency to the letter h. Subject M7 produced inward palm orientation on the letter c in the words “school” (misspelled “shchool”) and “teach” (produced as “teteach”), on the letters o-n-e in the word “phone” (spelled “pphone”), on the letter a in the word “chair,” and on the letters t and e in the word “mother.” In two instances, the occurrence of h appears to have triggered the spreading of inward-facing orientation across the rest of the word; both M7’s production of “phone” and M8’s production of “chair” (misspelled “chardir”) contained reversals on each of the fingerspelled letters that occurred subsequent to the h.

The opposite effect also appeared in our data: rather than the spreading of inward-facing palm orientation onto segments that are typically produced with outward-facing orientation, we also observe the spreading of outward-facing orientation onto segments that are typically produced with inward-facing orientation. These examples include the h in “phone” produced by subject M1, the g in “girl” produced by subject M2, the g in “girl” (misspelled “gierl”) by subject M10, and the h in “father” produced by subject M10. We also find two instances of word-final reversals: the r in “mother” (misspelled “moter”) by subject F4 and the r in “paper” produced by subject M7.

Importantly, there were at least 64 reversal errors produced by children with ASD that cannot be explained by adjacency. Some words that did not contain g, h, p or q nonetheless contained reversal errors: these included the v in “van” produced by subject M2, all four letters in the word “desk” produced by subject M8, each letter in the word “table” (misspelled “tadile”) by subject M8, all three letters in the word “van” produced by subject M8, all four letters in the word “ball” produced by subject M17, all four letters in the word “bird” produced by subject M17, each of the letters except the initial letter in “desk” (misspelled “deask”) by subject M17, and each of the letters in the word “table” (misspelled “tabitable”) by subject M17.

### Longitudinal data

This study included three participants whose fingerspelling had been analyzed in Shield and Meier’s (2012) preliminary study on fingerspelling. Comparing their performance in this study to the previous study is instructive insofar as it can provide additional information about the developmental trajectory of palm orientation reversals. See Table 7 for information about the ages at which these three participants were tested, intelligence, language, and ASD severity scores, as well as proportion of reversed fingerspelled letters in both studies.

**Table 7:** Longitudinal analysis: three children participated in Study 1 (Shield and Meier, 2012) and Study 2 (the current study).

Subject ID Study 1/Study 2	Study 1: Age	Study 1: No. of finger-spelled letters with reversed palm	Study 2: Age	Study 2: No. of finger-spelled letters with reversed palm	Study 2: NVIQ SS	Study 2: ASL RST SS	Study 2: ADOS severity
Child 1/M8	7;5	27/57 (47.4%)	12;7	34/79 (43.0%)	96	81	6
Child 3/M7	6;6	26/43 (60.5%)	10;2	10/75 (13.3%)	69	70	6
Child 4/M4	5;8	19/28 (67.9%)	9;8	0/69 (0%)	117	79	6

While all three of these participants produced reversals in their fingerspelling data in the prior study (Shield and Meier, 2012), only two, M7 and M8, continued to do so in this study. Subject M8 produced a similar proportion of fingerspelled letters with reversed orientation at both ages, while Subject M7 produced fewer letters with reversed palm orientation in this study (10/75) than in the earlier study (26/43). Subject M4, who produced 19 reversed-orientation letters in the earlier study, no longer produced any palm orientation reversals in this study.

## Discussion

The purpose of this study was to explore the variation in palm orientation of fingerspelled letters produced by native-signing children with and without ASD. Once we identified which children with ASD produced palm reversal errors, we analyzed and compared their cognitive and linguistic profile to that of the children with ASD who did not frequently produce palm reversal errors as well as to a control group of TD deaf children.

As expected, signing children with ASD produced significantly more palm reversal errors than TD signing children. Overall, the participants with ASD produced palm reversal errors on an average of 6.7% of fingerspelled letters, a palm reversal error rate much lower than that found by Shield and Meier’s (2012) fingerspelling study, which reported a reversal rate of 40.2% by four native-signing children with ASD. The current study included a much larger sample of signing children with ASD (*N* = 17), which may be more representative of the overall population of signing children with ASD. The TD participants in our study produced palm reversal errors on just 0.6% of fingerspelled letters overall, a significantly lower rate than that of the participants with ASD. Most of the palm reversals produced by the TD participants could be attributed to idiosyncratic individual factors (for the u-h substitutions produced by TD participant F7) or to phonetic context, whereas many of the palm reversals produced by the ASD group could not be explained by either of these factors. Only a subset of participants with ASD (*n* = 7) produced one or more palm reversal errors, with individual reversal rates of these participants ranging from 1.3 to 46.7% of all letters produced. Three of the children with ASD in particular accounted for the preponderance of palm reversal errors in the ASD group (79/87 errors). The five participants who reversed two or more fingerspelled letters were found to have significantly lower receptive language abilities in comparison to the participants with ASD who produced one or no palm reversal errors. There were no statistically significant differences between the reversers and non-reversers with respect to age, ASD severity, theory of mind, or visual perspective-taking skills.

### Comparison of palm reversals to pronoun reversals

One goal of this study was to compare the cognitive and linguistic profile of the children with ASD in this study who produce palm reversal errors to the cognitive and linguistic profile of hearing children with ASD who produced pronoun reversals as reported in previous literature. We posited that the two error types have a fundamental similarity in that the forms produced by children involve a type of wholesale or gestalt reproduction of the linguistic form (e.g., production of the word “you” in reference to self in the case of pronoun reversals; production of inward-facing palm rather than outward-facing palm, or vice versa, in the case of palm reversals). We thus speculated that a difference in learning/imitation style in very young TD children and children with ASD could result in different surface phenomena in signed and spoken languages.

Further, we asked if there was evidence that both phenomena occurred:

(a) at similar chronological ages (for both typical and atypical children);
(b) at similar frequencies and with similar (in)consistency within the population of children with ASD;
(c) in children with similar linguistic and/or cognitive profiles, and/or
(d) in individual children with ASD who are bimodal bilinguals in a signed language and in a spoken language.

With regard to point (a), our sample did not include TD children in the age range at which pronoun reversals are reported in the literature (under the age of 2;6). In our sample of signing children with and without ASD, palm reversals were produced throughout the school-age years, with the oldest reverser being 12;6. This included several TD signing children who produced palm reversals, though only one TD child produced more than one reversal error, and this was produced consistently on the letter h and did not spread to other segments. With regard to the children with ASD, the age at which children produced palm reversals is similar to the ages at which hearing children with ASD are reported to produce pronoun reversals in the literature. In particular, several studies on hearing children with ASD have reported pronoun reversals persisting into adolescence (Jordan, 1989; Lee et al., 1994). However, we also find evidence that palm reversals disappear for some children over time: one of the three children who was studied by Shield and Meier (2012) and who produced palm reversals in that study no longer produced palm reversals in the current study, 4 years later. These findings align with the literature on pronoun reversals, which suggests that some children with ASD stop reversing pronouns as development progresses (Kanner, 1943; Naigles et al., 2016).

With regard to point (b), the overall rate of palm reversals in our study (6.7% of fingerspelled letters produced by the children with ASD) is not far from the rate of pronoun reversals produced by speaking children with ASD in some studies in the literature. For example, Naigles et al. (2016) reported a pronoun reversal rate by toddlers with ASD of 7.07% at visit one (when mean age was 31.6 months), averaging 4.15% across all six visits lasting 2 years. Like pronoun reversals, palm reversals are produced inconsistently, even by the children we have labeled as “reversers”; none of the children in our study consistently reversed palm orientation on all fingerspelled segments. As was also reported by Shield and Meier (2012) and Shield et al. (2020), participants with ASD who exhibited a pattern of palm reversal errors did so inconsistently across word contexts. For example, participant M17 in the ASD group produced palm reversal errors on both Ls in the word *ball*, but accurately produced both Ls in the word *doll* with outward palm orientations later in the fingerspelling task. This, too, mirrors the literature on pronoun reversal: hearing children with ASD inconsistently reverse pronouns, such as the six participants in Tager-Flusberg’s (1994) study who reversed 13.2% of all of the pronouns in the sample.

Also with regard to point (b), it is clear that palm orientation reversal errors, like pronoun reversals, are produced by a subset of children of ASD. In our sample of native-signing children with ASD, five of the 17 children (29.4%) produced more than one palm reversal (and two additional children produced one palm reversal each, for a total of 41.2% of the sample). The literature reports a wide range of proportions of hearing children with ASD who produce pronoun reversals (2.6%: Barokova and Tager-Flusberg, 2020; 12%: Lee et al., 1994; 27.3%: Jordan, 1989; 63.6%: Kanner, 1943). What is consistent is that it is never the case that every child with ASD within a sample produces pronoun reversals, and our results echo that finding.

With regard to point (c), our study found that palm reversal was most strongly associated with lower receptive language skills within the ASD group, but not within the TD group. There are some resonances between our finding and the literature on pronoun reversals in hearing children with ASD. For example, Naigles et al. (2016) reported that their participants with ASD who produced pronoun reversals had lower vocabulary and joint-attention scores than the participants with ASD who did not produce pronoun reversals. Similarly, the participants in Jordan’s (1989) study demonstrated impaired language abilities, with a mean MLU of 2.4 and expressive vocabulary abilities with an age equivalent of 5;7 (despite having an average chronological age of 10;5), as well as intellectual disability, with a mean IQ of 49. The six participants in Tager-Flusberg’s (1994) study, too, had an average MLU of 2.24 despite being between the ages of 3 and 10 years old, indicating impaired language abilities.

Finally, with regard to point (d), we did not study the spoken language development of any of the children in our sample, so we cannot comment on whether or not they may produce pronoun reversals in spoken English.

In summary, it seems that hearing children with ASD who produce pronoun reversal errors in their speech tend to exhibit impaired language and/or impaired social cognition. Likewise, the participants with ASD who produced palm reversal errors in our study tended to have lower receptive language abilities when compared to their non-reversing peers. However, there was no significant difference in measures of social cognition between the reversers and non-reversers, at least among the children with ASD (though note that the TD group was significantly better on measures of VPT and ToM). Therefore, at this time, there is not sufficient evidence to support the hypothesis that deficits in social abilities such as ToM could be underlying palm reversal, as was found for pronoun reversal by Naigles et al. (2016).

Nonetheless, pronoun reversals and palm reversal errors appear to share the following characteristics:

- Both error types could reflect a “gestalt” learning style in which children (re)produce linguistic forms without undergoing requisite shifts.
- Both error types are produced more frequently by children with ASD than TD children.
- Both error types are produced by a subset of children with ASD, not all children with ASD.
- Both error types can be produced by children with ASD into (at least) adolescence.
- Both error types may follow a developmental trajectory and disappear over time, for at least some children.
- Both error types are produced relatively infrequently overall.
- Both error types are produced inconsistently by the children who produce them.
- Both error types seem to be associated with impaired language skills within the population of children with ASD.

These similarities are certainly suggestive of parallel phenomena. However, it would be premature to definitively state that palm reversal errors and pronoun reversal errors are analogous phenomena in two different language modalities, for reasons that are explained in the next section.

### Limitations and suggestions for future research

While this study documented a number of similarities between pronoun reversals in speech and palm reversals in sign, there are needed pieces of evidence that are now missing. For example, there is no strong evidence in the literature for palm reversals produced by very young TD deaf children at the ages at which pronoun reversals typically occur in hearing, speaking children (i.e., under the age of 2;6). Indeed, the palm orientation parameter is typically acquired rather early on, especially when compared to the more difficult handshape and movement parameters (Cheek et al., 2001).

Similarly, there is currently only one report of two possible pronoun reversal errors in signers with ASD (Shield et al., 2015), despite a few reports of pronoun reversals produced by four TD signers at very young ages (Petitto, 1987; Jackson, 1989; Pizzuto, 1990). The documentation of pronoun reversals by these young signers would suggest that they may also occur in older signers with ASD. Future studies should continue to document the use of sign-language pronouns by signers with ASD into the school-age years and adolescence. To-date, there is only one report on the use of sign-language pronouns by signers with ASD (Shield et al., 2015); this study found avoidance of pronouns in favor of sign-names or common nouns, but did not document any pronoun reversals.

Future research should further explore the relationships between palm reversal and other aspects of social cognition. While this study found that reversers had lower receptive language skills than non-reversers, there was no strong relationship with difficulties in social cognition, such as in ToM or VPT. Studies of younger deaf children with ASD should document early joint-attention skills in relation to sign-language development in order to better understand how these skills may be related.

The finding of phonetic contexts that may condition palm reversals (such as adjacency to the letters g, h, p, and q) is unlike anything that has been documented for pronoun reversals in spoken languages. Since palm reversals are a phonetic phenomenon involving one of the parameters of sign articulation, the orientation of the palm can spread to neighboring segments. By contrast, pronouns are individual lexical items, and pronoun reversal involves the substitution of lexical forms rather than phonological values. Even if it is discovered that both phenomena are linked to the same underlying processes, we would not expect the phenomena to behave in exactly the same way, since they function in different areas of language. Relatedly, the cognitive demands of fingerspelling are likely to be quite different from those of producing pronouns in spoken languages, since fingerspelling is tied to letter recognition and literacy. Although working memory is presumably not a constraint on performance in this task (given that participants could view the printed stimulus throughout each trial), children must recognize the printed letter, retrieve the correct fingerspelling handshape from long-term memory, and produce the fingerspelling handshapes in left-to-right order. Indeed, we found a relationship [*r* (39) = 0.33, *p* < 0.05] between fingerspelling accuracy and palm reversal errors, suggesting that it is possible that palm reversals are largely observed in fingerspelling because fingerspelling places a relatively higher cognitive load on signers than does the production of lexical signs.

Our study was limited to just one aspect of ASL: fingerspelling of English words. Fingerspelling was explored due to the fact that it is an area that has previously been shown to reveal difficulties with palm orientation (e.g., Shield and Meier, 2012; Shield et al., 2020); however, fingerspelling is but a small part of the overall linguistic system of ASL. In comparing the rates of palm reversal errors in our participants with the rates of pronoun reversal errors in the literature on hearing children with ASD, readers are cautioned to take this fact into account.

One particularly promising route for future research could involve bimodal bilinguals with ASD. These are children who are acquiring a signed language and a spoken language simultaneously. It would be particularly compelling, for example, if such children produced pronoun reversals in speech at the same time that they exhibited palm orientation reversals in sign. To date, there are no reports on the signed- and spoken-language development of bimodal bilinguals with ASD (though the longitudinal case study reported by Shield et al. (2020) focused on the signed-language development of a hearing child of Deaf adults). Although this child is a bimodal bilingual, Shield et al. only analyzed his signing (not his speech), so this study does not shed light on whether or not pronoun reversals in speech and palm reversals in sign co-occur in the same individuals. It is also worth noting that two of the three children with ASD who produced the majority of the palm reversals were hearing bimodal bilinguals (M7: 10 reversal errors; M17: 35 reversal errors). Although we do not have reason to believe that the hearing status of these children influenced their production of palm reversals, future research should consider whether the hearing children of Deaf adults may be more susceptible to reversal errors than deaf children of Deaf parents.

## Conclusion

We have presented a study in which we compared palm reversal errors in the fingerspelling of signing children with and without ASD to the phenomenon of pronoun reversals produced by hearing children with and without ASD. There is no question that the two phenomena present some tantalizing similarities which merit more study in the future. Should the two phenomena be more convincingly found to be analogous, they would constitute an interesting example of how the cognitive and social characteristics of ASD yield different linguistic behaviors in the signed- versus spoken-language modalities.

## Data availability statement

The original contributions presented in the study are included in the article/supplementary material, further inquiries can be directed to the corresponding author.

## Ethics statement

The studies involving human participants were reviewed and approved by Boston University Institutional Review Board. Written informed consent to participate in this study was provided by the participants’ legal guardian/next of kin.

## Author contributions

AS designed the study and collected and analyzed the data. MI coded the data and wrote the first draft of the manuscript. AS and RM revised the manuscript. All authors contributed to the article and approved the submitted version.

## Funding

This work was supported by grant 1F32-DC0011219 from NIDCD and Research Enhancement Grant 14-04 from the Autism Science Foundation to AS. Publication costs were provided for by the Office of Research at Innovation at Miami University and the Robert D. King Centennial Professorship of Liberal Arts at the University of Texas at Austin.

## Conflict of interest

The authors declare that the research was conducted in the absence of any commercial or financial relationships that could be construed as a potential conflict of interest.

## Publisher’s note

All claims expressed in this article are solely those of the authors and do not necessarily represent those of their affiliated organizations, or those of the publisher, the editors and the reviewers. Any product that may be evaluated in this article, or claim that may be made by its manufacturer, is not guaranteed or endorsed by the publisher.

## Footnotes

^1^Kanner’s report included the following information about his subjects’ uses of pronouns: Alfred L. "confuse[d] pronouns" (p. 233) at age three-and-a-half; Charles N.’s "vocabulary [wa]s good, except for pronouns" (p. 236) at age four-and-a-half; John F. "used the pronoun of the second person when referring to himself" (p. 238) until age four-and-a-half, when he "began gradually to use pronouns adequately" (p. 238); Elaine C. at age five "did not use pronouns correctly" (p. 240) and at age 7;2 "never use[d] the personal pronouns of the first and second persons correctly" (p. 241); Paul G. made pronoun errors at age five ("all statements pertaining to himself were made in the second person," p. 228); Donald T. reversed pronouns in echolalic contexts at ages 5;1–5;5, but no longer did at age 7;7; and Frederick W. "ha[d] great difficulty in learning the proper use of personal pronouns" (p. 223) at age six.

^2^Note that these letters could have been coded as instances of the letter u without palm reversal. In this case, these would constitute errors in the accuracy of representing the printed word, but would not have constituted palm-reversal errors. We have chosen to code these as instances of the letter h, rather than u, due to the consistency of the error.
